# A novel method to determine antibiotic sensitivity in *Bdellovibrio bacteriovorus* reveals a DHFR-dependent natural trimethoprim resistance

**DOI:** 10.1038/s41598-020-62014-x

**Published:** 2020-03-24

**Authors:** Emanuele Marine, David Stephen Milner, Carey Lambert, Renee Elizabeth Sockett, Klaas Martinus Pos

**Affiliations:** 1 Institute of Biochemistry, Goethe-University Frankfurt, D-60438 Frankfurt am Main, Germany; 2 Institute of Immunology and Microbiology, School of Life Sciences, University of Nottingham, Queen’s Medical Centre, Nottingham, NG7 2UH UK; 3 Present Address: Department of Zoology, University of Oxford, Oxford, OX1 3SZ UK

**Keywords:** Enzyme mechanisms, Antimicrobials

## Abstract

*Bdellovibrio bacteriovorus* is a small Gram-negative bacterium and an obligate predator of other Gram-negative bacteria. Prey resistance to *B. bacteriovorus* attack is rare and transient. This consideration together with its safety and low immunogenicity makes *B. bacteriovorus* a valid alternative to antibiotics, especially in the treatment of multidrug resistant pathogens. In this study we developed a novel technique to estimate *B. bacteriovorus* sensitivity against antibiotics in order to make feasible the development and testing of co-therapies with antibiotics that would increase its antimicrobial efficacy and at the same time reduce the development of drug resistance. Results from tests performed with this technique show that among all tested antibiotics, trimethoprim has the lowest antimicrobial effect on *B. bacteriovorus*. Additional experiments revealed that the mechanism of trimethoprim resistance in *B. bacteriovorus* depends on the low affinity of this compound for the *B. bacteriovorus* dihydrofolate reductase (Bd DHFR).

## Introduction

**Figure 1:**
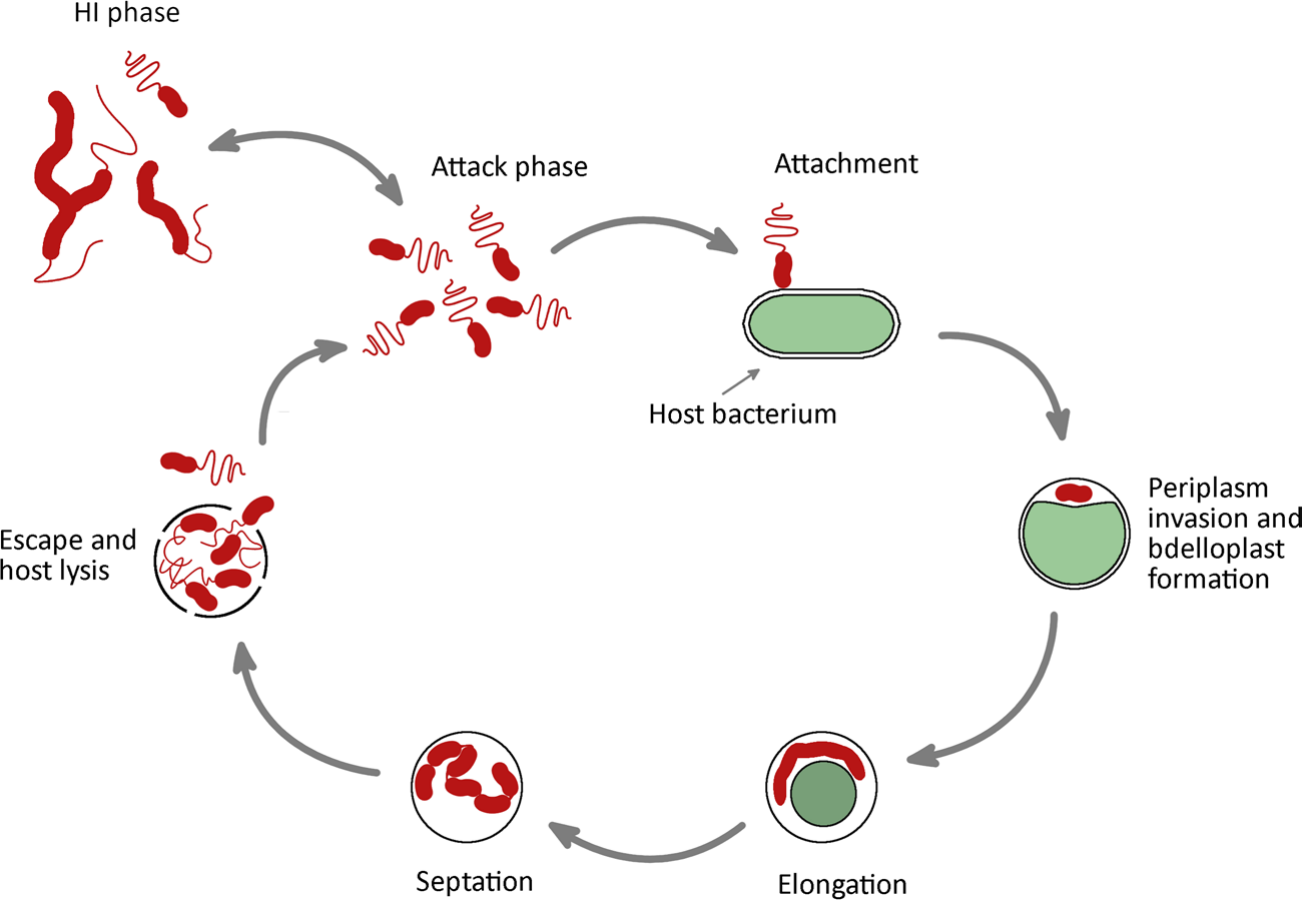
*B. bacteriovorus* predatory life cycle.

The increasing development and spread of antibiotic resistant bacteria is an ever-rising problem worldwide with an alarming rise of cases involving Gram-negative bacteria^1^. Bacteria can transfer their acquired resistance to other bacteria and can accumulate multiple resistance genes giving rise to multiple drug resistance (MDR)^2^. On the other hand, the rate of development and approval of new antibiotics is dramatically declining, mostly because of economic reasons due to the higher standards required by western medicine agencies and the higher profitability of other pharmaceutical classes. In light of this, the use of different pre- and pro-biotic agents as alternatives to antibiotics is considered an appealing option^3,4^. Predatory bacteria are regarded as one of the most promising possibilities to treat MDR pathogens. *Bdellovibrio bacteriovorus* belongs to the *Bdellovibrionaceae* family and is one of the most extensively studied predatory bacteria. The life cycle (Fig. 1) is characterized by the periplasmic invasion of Gram-negative prey bacteria. It starts by the encounter and attachment of free-swimming (or attack phase) *B. bacteriovorus* to prey. Upon entry, the host bacterium rounds up and becomes metabolically inactive (this whole replicative unit of predator inside prey is named a bdelloplast). Then, *B. bacteriovorus* establish itself in the periplasm and undergoes filamentous growth. When all nutrients are depleted, it synchronously divides by septation, giving rise to multiple progeny, which grow flagella and escape by lysis of the host cell.

*B. bacteriovorus* efficiently prey upon bacteria organized in biofilms^5,6^ and other structures such as polymicrobial infections in cystic fibrosis patients^7^. Furthermore, *B. bacteriovorus* appears to be effective in preying upon numerous Gram-negative pathogens, such as MDR human pathogens including *Acinetobacter baumannii*, *Escherichia coli*, *Klebsiella pneumoniae* and *Pseudomonas aeruginosa*^8^. The main advantage of using *B. bacteriovorus* as a therapy is the inability of prey bacteria to develop resistance against predation. However, transient resistance has been observed and, akin to bacterial persistence, is lost on further prey bacterial replications^9,10^. As a consequence of this phenomenon, *B. bacteriovorus* predation while killing a large percentage of prey, *in vitro*, never results in complete eradication of the attacked population^11,12^, regardless of the initial predator to prey ratio (PPR) in the bacterial pool.

One possibility is to use *B. bacteriovorus* in combination with conventional antibiotics to potentiate the effectiveness of the antibiotic therapy against Gram-negative pathogens (including MDR cells within the population). In order to make this approach feasible more has to be known about the ability of *B. bacteriovorus* to resist antibiotics itself. Standard assays to determine the Minimum Inhibitory Concentration (MIC) are not suitable for *B. bacteriovorus*, since for growth, this species requires the presence of bacterial prey cells (which are themselves susceptible to antibiotics). The use of *B. bacteriovorus* in a co-therapy with antibiotics has already been proposed^9,13^. In a study by Im *et al*.^14^, *B. bacteriovorus* has been used successfully in combination with violacein, an antibiotic effective against Gram-positive bacteria, to treat MDR pathogens. The combination of both antibacterial agents allowed to expand their spectrum of action (that in case of *B. bacteriovorus* is restricted to Gram-negative bacteria) to treat Gram-positive and -negative mixed bacteria populations.

In this study, we developed a novel assay in liquid medium, which allows testing of *B. bacteriovorus* sensitivity against antibiotics. For this purpose, we tested antibiotics belonging to different classes of pharmaceuticals with different specificities for Gram-positive and -negative bacteria. *B. bacteriovorus* growth is estimated by the reduction in absorbance at 600 nm (caused by the lysis of prey cells) of mixed *B. bacteriovorus/E. coli* cultures in the presence and absence of different antibiotics concentrations. Antibiotic minimal inhibitory concentration (MIC) values are subsequently determined by non-linear regression. Our findings show that among all tested antibiotics, trimethoprim (TMP) is the least active on *B. bacteriovorus*.

The most common mechanism of natural resistance against TMP is caused by the lack of affinity of the dihydrofolate reductase (DHFR) for this antibiotic compound. In order to ascertain whether this mechanism is the cause of resistance in *B. bacteriovorus*, we cloned three *B. bacteriovorus* genes (*bd3231*, *bd*0*323*, *bd1356*) with homologies to the *E. coli folA* chromosomal gene (*b*00*48*, encoding the DHFR enzyme) and analyzed their sensitivity against TMP by plate dilution assays. By this screening we identified the *B. bacteriovorus* DHFR (Bd DHFR) as the protein encoded by the *bd3231* gene. The involvement of certain amino acids (V6, M29 and F51) in TMP binding is also investigated by site-directed mutagenesis, plate dilution and MIC assays. The kinetic properties and TMP inhibition of activities are studied on the purified protein and its substituted variants.

## Results

### Determination of *B. bacteriovorus* antibiotic sensitivity

**Figure 2:**
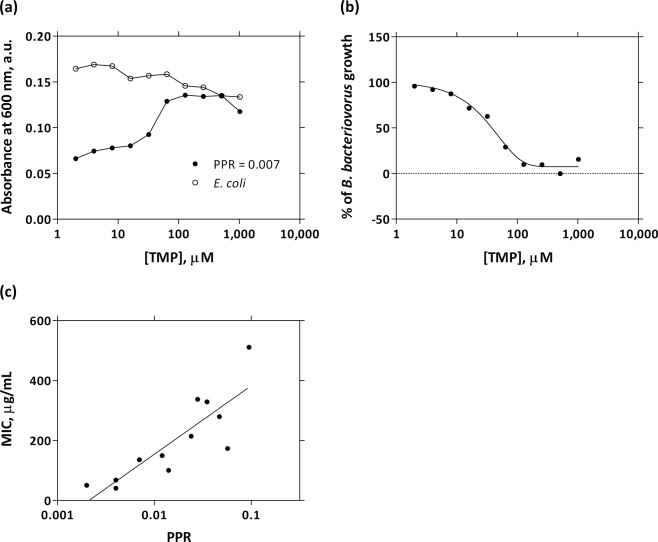
MIC determination of TMP for *B. bacteriovorus*. (**A**) OD_600_ values of *B. bacteriovorus*/*E. coli* mixtures (initial predator to prey ratio (PPR) of 0.007) measured at different TMP concentrations. The series labeled as *E. coli* relates to the control (initial PPR equal to 0). (**B**) Absorbance Δ values expressed in percentage of *B. bacteriovorus* growth. The MIC value is calculated by fitting growth data to a modified Gompertz equation^15^. (**C**) Estimation by linear regression of the theoretical TMP MIC for *B. bacteriovorus* at the PPR of 0.02.

*B. bacteriovorus* is a relatively small-sized bacterium (0.25–0.35 by 0.5–2.5 μm) and therefore does not scatter light efficiently at 600 nm. Direct cell growth measurements of *B. bacteriovorus* upon prey bacteria can be estimated by reduction of the optical density at 600 nm (OD_600_) (i.e. the loss of culture turbidity by *B. bacteriovorus*-induced lysis of prey cells). In this study, we designed a 96-well based method for the determination of antibiotic MICs for *B. bacteriovorus*. *B. bacteriovorus* HD100 cells were added to an *E. coli* culture and the reduction of OD_600_ measured in absence and presence of different concentrations of antibiotics. Assays were conducted in a solution of Ca/HEPES buffer and the exhausted medium transferred with the *E. coli* overnight culture and the *B. bacteriovorus* dilution. Therefore, *E. coli* cells were in the stationary phase and their growth during the assays was negligible. In Fig. 2a is an example of the MIC value determination for TMP (results from a single PPR series). MIC values are dependent on the initial PPR used in the assay, as standard MIC assays are dependent on the initial bacterial concentration. However, *B. bacteriovorus* numbers and therefore, the initial PPR can be estimated reliably only after the assay by plating in double layer agar plates. The relation between initial PPR and MIC values was then investigated by adding different numbers of *B. bacteriovorus* cells to the fixed number of *E. coli* prey cells in parallel replicated experiments (on different rows of the same plate containing replicated antibiotic serial dilutions). We also considered the effect of antibiotics on the *E. coli* prey cells and measured the OD_600_ values of *E. coli* in the absence of *B. bacteriovorus* (PPR = 0), at the same antibiotic concentrations. *B. bacteriovorus* growth is estimated by subtracting absorbance values of *B. bacteriovorus* at different PPRs from the values of *E. coli* (PPR = 0) at the same antibiotic concentrations. Therefore, for each PPR series of data is extrapolated a Δ curve of *B. bacteriovorus* growth, in which the 100% growth refers to absorption values of samples not treated with antibiotic (corresponding to the highest level of prey lysis that is achieved at that particular initial PPR). The efficacy of an antibiotic on *B. bacteriovorus* is displayed by the lack of *E. coli* lysis. In order to determine the MIC of an antibiotic, *B. bacteriovorus* growth data at different PPRs (plotted against the logarithm of antibiotic concentration) were fitted with a modified Gompertz equation^15^ (Fig. 2b). Antibiotic MIC values were extrapolated from each curve as the minimal antibiotic concentration that prevents *B. bacteriovorus* growth. The theoretical MIC value was calculated for all antibiotics by linear regression at a PPR of 0.02 in a plot of MIC values against the logarithm of initial PPRs (Fig. 2c).

### Comparison between *B. bacteriovorus* and *E. coli* resistance to antibiotics

The MIC values obtained for *B. bacteriovorus* are not comparable with the values from standard MIC assays for other bacteria due to the different conditions employed in this method. We therefore determined the MIC values of selected antibiotics for *E. coli* as close as possible to the same conditions used in the *B. bacteriovorus* assay (since *E. coli* does not feed on prey bacteria). The higher the difference between the MIC value of a given antibiotic for *B. bacteriovorus* in comparison to *E. coli* (ratio»1), the more effective would be a *B. bacteriovorus* based co-therapy against *E. coli*. Antibiotics characterized by a high *B. bacteriovorus*/*E. coli* MIC ratio, such as amikacin, kanamycin, gentamicin and aztreonam (Table 1), are of particular interest for a *B. bacteriovorus* based co-therapy against *E. coli* infections. At the bottom of Table 1 are listed the antibiotics for which it was not possible to measure the MIC for *E. coli* under the same conditions (i.e. TMP) or that are not active against *E. coli* (i.e. lincomycin, spiramycin and vancomycin). In particular, TMP MIC could not be determined due to the presence of thymine and thymidine in the culture broth (Ca/HEPES:YPSC, Table S1). However, TMP gained our attention as it is the antibiotic characterized by the highest MIC for *B. bacteriovorus* with a value equal to 223 ± 24 µg/mL.

**Table 1:** Antibiotic MIC values (µg/mL) for *E. coli* BW25113 (MIC *Ec*) and *B. bacteriovorus* HD100 (MIC *Bd*).

Antibiotic	MIC *Bd*	MIC *Ec*	MIC *Bd*/*Ec*
Amikacin	0.70 ± 0.02	0.060	12
Kanamycin	0.69 ± 0.06	0.081	8.6
Gentamicin	0.06	0.0084	7.2
Aztreonam	41 ± 4	5.7	7.2
Levofloxacin	0.23 ± 0.02	0.064	3.7
Meropenem	0.42 ± 0.06	0.14	2.9
Ciprofloxacin	0.31 ± 0.04	0.13	2.4
Cefoperazone	1.3 ± 0.3	0.60	2.1
Cefaclor	18 ± 2	10	1.7
Ceftazidime	7.8 ± 0.4	19	0.42
Tetracycline	1.7 ± 0.2	4.8	0.35
Tigecycline	0.59 ± 0.01	2.3	0.26
Cefazolin	0.51 ± 0.07	2.0	0.25
Ampicillin	1.3 ± 0.2	5.4	0.25
Penicillin G	3.4 ± 0.5	19	0.18
Piperacillin	2.5 ± 0.5	122	0.021
Chloramphenicol	0.16	10	0.016
Lincomycin	7.9 ± 0.9		
Spiramycin	8.3 ± 1.9		
Trimethoprim	223 ± 24		
Vancomycin	16 ± 1		

Antibiotics are listed by the degree of resistance of *B. bacteriovorus* over *E. coli* (ratio between MIC values; MIC *Bd/Ec*). MIC values for *B. bacteriovorus* are expressed as mean ± standard error.

### Bd3231 DHFR confers TMP resistance to *E. coli*

**Figure 3:**
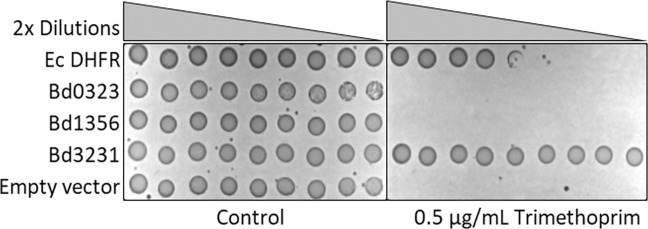
Expression of the *B. bacteriovorus* DHFR (Bd3231) in *E. coli* confers resistance against TMP. *E. coli* BW25113 expressing either Bd0323, Bd1356, Bd3231 or Ec DHFR were cultivated overnight. Two-fold dilutions of cells were spotted onto MHII 1.5% (w/v) agar plates supplemented with 0.01 mM IPTG and either 0 or 0.5 μg/mL TMP.

The known target of TMP is the dihydrofolate reductase (DHFR), an enzyme that catalyzes the reduction of 7,8-dihydrofolate (DHF) to 5,6,7,8-tetrahydrofolate (THF) by using nicotinamide adenine dinucleotide phosphate (NADPH) as cofactor^16–19^. This reaction is essential for the *de novo* synthesis of purines and some amino acids. A common mechanism of resistance against TMP is the lack of affinity of this compound for DHFR. To test whether the *B. bacteriovorus* DHFR (Bd DHFR) is insensitive towards TMP, we cloned three *folA* homologs from *B. bacteriovorus* (*bd3231*, *bd0323*, *bd1356*) in *E. coli* and analyzed them by plate dilution assays (Fig. 3). The *B. bacteriovorus* gene *bd3231* confers very high resistance against TMP, even exceeding the resistance caused by the recombinant *E. coli folA*, despite its apparent two-fold higher expression levels (Figs. S1 and S2). The other *B. bacteriovorus* genes were expressed at levels of apparent 1.3-fold the *E. coli folA* expression, however, did not confer a TMP-resistant phenotype. Thus, we named Bd3231 as Bb DHFR.

### Role of selected amino acids in TMP binding to Bd DHFR

**Figure 4:**
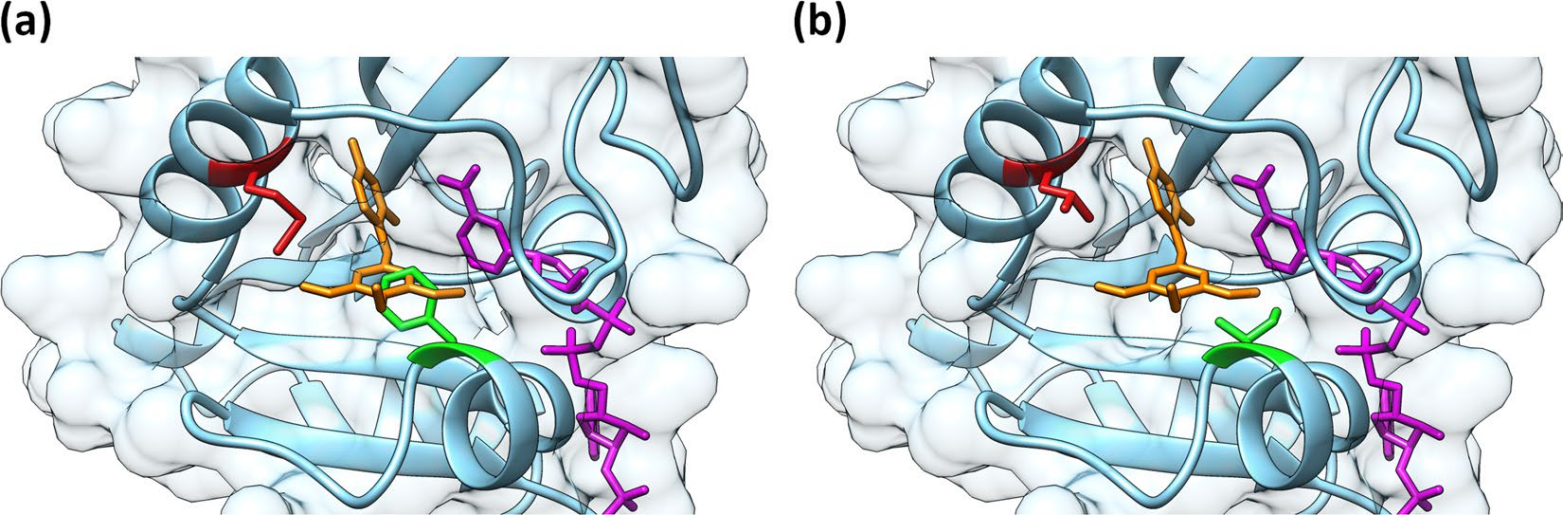
Postulated TMP binding to Bd DHFR wild type (**A**) and Bd DHFR_M29L_F51I (**B**) catalytic site, based on Phyre2 homology modeling^30^ using *C. burnetii* DHFR (PDB entry: 3TQ8^21^) as template. Bd DHFR structures are visualized in light blue, the residues at position 29 and 51 in red and green, respectively. TMP (orange) and NADPH (purple) coordinates are obtained by Bd DHFR superposition with the DHFR complex structure of *C. burnetii*. Molecular graphics and analyses were performed with UCSF Chimera (Version 1.11)^36^, developed by the Resource for Biocomputing, Visualization, and Informatics at the University of California, San Francisco, with support from NIH P41-GM103311.

**Figure 5:**
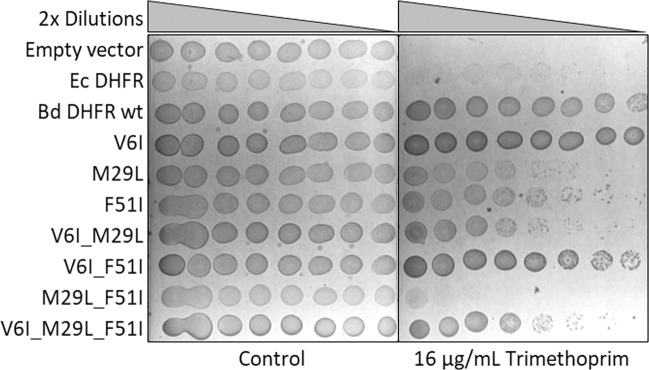
TMP resistance screening of Bd DHFR substituted variants. *E. coli* MC1061 expressing either Bd DHFR, a substituted variant or Ec DHFR were cultivated overnight. Two-fold dilutions of cells were spotted onto MHII 1.5% (w/v) agar plates supplemented with 0.05% L-arabinose and either 0 or 16 μg/mL TMP.

**Figure 6:**
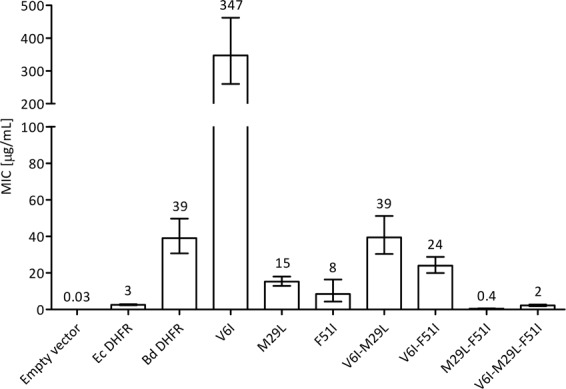
TMP MIC values for *E. coli* expressing Bd DHFR substituted variants. Measurements were performed in 96-well plates as quadruplicates, using MHII broth supplemented with 50 µg/mL kanamycin and 0.01 mM IPTG. Plates were incubated at 37 °C for 20 hours under shaking conditions. MIC values were calculated by fitting data to a modified Gompertz equation^15^. Error bars represent 95% confidence intervals.

We deduced from a DHFR amino acid sequence alignment of TMP sensitive vs. resistant bacteria, in combination with the structural insights from binding of TMP to the *E. coli* DHFR^20^ (Ec DHFR; Fig. S3, Table S2) and *Coxiella burnetii* DHFR (PDB: 3TQ8^21^; Fig. 4), that three residues (V6, M29, and F51) might be responsible for the TMP-resistance observed for the Bd DHFR. To further analyze this hypothesis, we single-substituted these three residues in the Bd DHFR with the residues present in the TMP-sensitive DHFRs. These mutant genes were subsequently expressed in *E. coli* and tested for their sensitivity against TMP by means of plate dilution and MIC assays. Results show that the single substitutions F51I and M29L cause a decrease in the resistance against TMP (Figs. 5 and 6), suggesting that TMP might have a higher affinity for these DHFR variants and therefore be able to inhibit their activity. With regard to the mutation V6I, TMP resistance levels appear to significantly increase in all Bd DHFR variants carrying this substitution (i.e. V6I, V6I_M29L, V6I_F51I and V6I_M29L_F51I; Figs. 5 and 6), possibly due to the higher expression of the mutant genes (Figs. S4 and S5). Double and triple substituted mutants show a consistent pattern in relation to the single and double substituted Bd DHFR variants bearing the wild type V6 amino acid.

The M29L and F51I substitutions confer a lower level of resistance, a strong indication that these amino acid residues could be involved in the TMP resistance mechanism. However, the higher expression of the M29L and V6I_M29L DHFR substitution variants might be an alternative explanation for the observed correlated resistance pattern towards TMP (Fig. S4). In order to analyze the effects of the substitutions on TMP binding independent of the expression levels, we overproduced and purified the wild type and substituted DHFR variants and conducted kinetic and TMP inhibition measurements.

### DHFR *in vitro* assays

In this study we developed a new plasmid, pBXKCPD, which is compatible with the FX-cloning method^22^ and allows to express target proteins with a His_10_-tagged CPD^23^ as C-terminal fusion (Fig. S6). This system allowed us to optimize and enhance cloning, expression and purification procedures. The Bd DHFR wildtype, the substituted variants and the Ec DHFR were overproduced and purified by Ni-NTA affinity chromatography and their purification tag removed by auto-catalytic cleavage (the Bd DHFR wild type was further subjected to size-exclusion chromatography).

The kinetic analysis of the different DHFR variants (Bd DHFR wild type, Ec DHFR, Bd DHFR_M29L, Bd DHFR_F51I and Bd DHFR_M29L_F51I) shows that at high substrate concentrations, a reduction in the initial velocity is apparent and describes a mechanism known as substrate inhibition (Fig. S7). In the Ec DHFR at high substrate concentrations, this mechanism involves the formation of the two dead-end complexes DHFR-DHF-NADP^+^ and DHFR-DHF-THF^16^.

Results from TMP inhibition assays (Table 2, Fig. S8) show that the Bd DHFR, with a *K*_*i*_ of 49 ± 15 nM, is 2,450 times more resistant against TMP inhibition than Ec DHFR (*K*_*i*_ of 0.02 nM^24,25^). Furthermore, it is evident that both the M29L and F51I substitutions cause a decrease in affinity for TMP, leading to a more inhibitor-sensitive enzyme. This is particularly evident in the double mutant, Bd DHFR_M29L_F51I, which has a *K*_*i*_ equal to 3.2 ± 1.6 nM, over 15 times lower than the wild type variant. The results from this analysis corroborates the results obtained from agar plate dilution and MIC experiments of *E. coli* expressing DHFR substituted variants. In light of this, we can conclude that the TMP sensitive phenotype observed in *E. coli* expressing M29L and F51I DHFR substituted variants is due to the enhanced TMP affinity of these DHFRs.

**Table 2:** Kinetic values and TMP inhibition constants for Bd DHFR wild type, its substituted variants and Ec DHFR. Values are expressed as mean ± standard error.

	Bd DHFR wt	Ec DHFR	Bd DHFR_M29L	Bd DHFR_F51I	Bd DHFR_M29L_F51I
*K*_*m*_ (µM)	5.6 ± 1.9	3.7 ± 2.4	3.4 ± 0.6	4.9 ± 1.1	9.2 ± 1.2
*K*_*cat*_ (s^−1^)	299 ± 21	10 ± 4	25 ± 18	73 ± 30	109 ± 7
*K*_*cat*_*/K*_*m*_ (M^−1^ s^−1^)	5.4·10^7^	2.6·10^6^	7.3·10^6^	1.5·10^7^	1.2·10^7^
TMP *K*_*i*_ (nM)	49 ± 15	0.02*	6.7 ± 1	15 ± 3	3.2 ± 1.6

*^24,25^.

## Discussion

### A novel method to determine antibiotic sensitivity in Bdellovibrio bacteriovorus

The method developed in this study allows to determine the degree of resistance of *B. bacteriovorus* against antibiotics relative to a reference organism (in this study *E. coli* BW25113). MIC values for antibiotics with *B. bacteriovorus* are prone to variation depending on the reference organism and growth conditions (i.e. culture broth, initial PPR, temperature, aeration and shaking force)^26^. The MIC values for both organisms were determined by fitting percentage growth values with a modified Gompertz equation^15^ rather than setting a threshold to absolute absorbance values. At some occasions, *B. bacteriovorus* growth percentage can show negative values (absorbance values of *B. bacteriovorus* cultures at different PPRs are higher than the values of *E. coli* controls (PPR = 0) at the same antibiotic concentrations), probably due to the presence of bdelloplasts and/or DNA of lysed cells in the bulk solution. This makes the use of a threshold for the determination of MIC values less reliable. On the other hand, by fitting growth data with the modified Gompertz equation, antibiotic MIC values are calculated as the concentrations at the interception between the lower asymptote lines and the tangent lines to inflexion points and are therefore independent from growth percentage values (Y axis).

This MIC method can be modified in order to test *B. bacteriovorus* against other pathogens and/or other antibiotics by changing the culture broth or the vessel to increase or reduce aeration according to requirements (e.g. 96-well plates rather than tubes or flasks). The main limitations of this technique are the use of non-standard conditions such as a culture broth containing Ca^2+^ and Mg^2+^ ions and a reduced temperature, which are required for *B. bacteriovorus* cultivation. Furthermore, this method may not allow for the determination of absolute MIC values of bactericidal antibiotics at concentrations equal or higher than their minimal bactericidal concentration (MBC). *B. bacteriovorus* is not able to replicate on heat-inactivated prey bacteria (with the exception of *B. bacteriovorus* host-independent strains^27,28^), while it can grow on UV-inactivated cells^29^. To the best of our knowledge, there are no studies regarding *B. bacteriovorus* ability to grow on antibiotic-inactivated cells. Therefore, prey bacteria inactivation at concentrations equal or above the MBC may prevent *B. bacteriovorus* from growing, resulting in a lower estimate of the MIC for *B. bacteriovorus* (this is true especially for lysed preys or preys with a compromised structural integrity).

The MBC values of bactericidal antibiotics are usually equal to, and generally do not exceed, four-fold the MIC values. On the other hand, the MBC values of bacteriostatic antibiotics are usually many folds over the related MIC values. Bacteriostatic antibiotics include macrolides, tetracyclines, sulfonamides, trimethoprim, chloramphenicol and linezolid, while bactericidal antibiotics include β-lactams, aminoglycosides, fluoroquinolones and vancomycin. Considering that MBC values are higher than the related MIC values, the determination of the MIC values for *B. bacteriovorus* may be underestimated only for those bactericidal antibiotics characterized by a MIC ratio (Bd/Ec; Table 1) above or equal to 1. However, whether prey bacteria are lysed or not is irrelevant for the determination of the Δ curve of *B. bacteriovorus* growth. Indeed, this curve is obtained by subtracting OD_600_ values of test samples (*B. bacteriovorus* at different PPRs) from OD_600_ values of *E. coli* control samples (PPR = 0) measured at the same antibiotic concentrations. Therefore, an eventual decrease in the OD_600_ caused by the activity of a bactericidal antibiotic will be measured in both control and test samples and will cancel out the difference between the two curves.

### Bdellovibrio bacteriovorus DHFR-dependent natural trimethoprim resistance

Our findings demonstrate that the *B. bacteriovorus* mechanism of resistance against TMP is caused by the low affinity of the DHFR for this compound, which is dependent on the presence of primarily two amino acid residues (i.e. M29 and F51) in the binding site. The Bd DHFR structure prediction analysis performed with Phyre 2^30^ using the *C. burnetii* DHFR (PDB entry: 3TQ8^21^; Fig. 4) as template suggests that M29 and particularly F51 may be in steric clash with TMP and/or reduce the cavity volume of the catalytic site disfavoring TMP binding. This hypothesis is supported by the substitution-induced increase in affinity of Bd DHFR for TMP by M29L and F51I. Both introduced side-chains are characterized by a reduced steric hindrance in comparison to the substituted residues, while their net charge and hydrophobicity are virtually unchanged.

Although the MIC method for *B. bacteriovorus* described in this work employs non-standard conditions, it allows for the first time to detect and compare *B. bacteriovorus* antibiotic resistance levels with other bacteria. In the perspective of employing *B. bacteriovorus* for co-therapies with antibiotics, its antibiotic resistance could be increased by insertion in the genome of resistance genes. This acquired resistance could be further investigated by the technique developed in this study and would allow the use of *B. bacteriovorus* in combination with further antibiotics. This study might pave the way for testing *B. bacteriovorus* (and other predatory bacteria) based co-therapies with antibiotics in both mammalian cell cultures and animals.

## Materials and Methods

### Construction of bacteria strains

All bacteria strains used in this study are listed in Table S3 of supporting information. Plasmids (Table S4) were constructed and genes cloned and/or modified as explained in the supporting information by using the primers listed in Table S5.

### *B. bacteriovorus* antibiotic resistance determination

*B. bacteriovorus* HD100 predatory growth was synchronized by sub-culturing into a YPSC (Table S1) *E. coli* BW25113 prey overnight culture (using a 2% inoculum), every 24 hours for 3 days as explained elsewhere^31^.

Two-fold serial dilutions of antibiotics (Table S6) were made along the rows of 96-well plates (BRANDplates pureGrade^TM^ S, no. 781662) in 100 µL Ca/HEPES buffer (5.94 g/L HEPES free acid, 0.284 g/L calcium chloride 2·H_2_O, adjusted to pH 7.6 with NaOH), including controls (no antibiotic present). *B. bacteriovorus* cultures were diluted by a factor of 25 in Ca/HEPES buffer. *B. bacteriovorus* two-fold serial dilutions were prepared in a new sterile 96-well plate along the column line (using as diluent a suspension of heat-inactivated *B. bacteriovorus*), including controls (no viable *B. bacteriovorus* present). Fifty µL of these dilution series were transferred from each well of the latter plate to the corresponding wells of plates with antibiotic dilution series. Subsequently, 50 µL of an *E. coli* BW25113 overnight culture grown in YPSC broth was added to each plate well. At this point, which corresponds to time zero, the blank was determined by measuring the OD_600_ of cell suspensions in a plate reader (TECAN Infinite 200 Pro). Final OD_600_ measurements were carried out after an incubation of 24 hours at 29 °C under shaking conditions (300 rpm, 3 mm throw). The number of *E. coli* cells was estimated by reading the OD_600_ of the overnight culture prior to the beginning of the assay. The number of viable *B. bacteriovorus* cells was estimated by plating in double layer YPSC agar plates as explained elsewhere^31^. Following an incubation time of three days at 29 °C, the number of *B. bacteriovorus* cells in the initial inoculation culture was estimated by counting plaque forming units (PFU). Enumeration of *B. bacteriovorus* by plaques count ensures that only viable cells are counted and, in our experience, is preferable over other methods based on microscopy and/or fluorescence. Pictures of plaques were acquired by the aid of a plaque visualization chamber, specially developed in this study for this purpose (Fig. S9).

All absorbance values were subtracted of the related blanks. Then, absorbance values of test samples (*B. bacteriovorus* co-cultures) were subtracted from the control values (*E. coli* without *B. bacteriovorus*) and plotted in graph against the logarithm of antibiotic concentrations. The resulting Δ curve is an indication of *B. bacteriovorus* growth. Data was expressed as percentage of growth and plotted in a graph of log(inhibitor) *vs*. response. The minimal inhibitory concentration (MIC) value was defined as the concentration of antibiotic that prevent *B. bacteriovorus* growth and were calculated by fitting *B. bacteriovorus* growth inhibition curves with a Gompertz modified equation^15^ (using GraphPad Prism 5). The theoretical MIC value for all antibiotics tested was determined by linear regression at a PPR of 0.02 in a plot of MIC values against the logarithm of initial PPRs.

### *E. coli* antibiotic resistance determination

Serial antibiotic dilutions in 100 µL Ca/HEPES were made along the rows of 96-well plates including controls without antibiotic. *E. coli* BW25113 cells from an overnight culture were washed and re-suspended in Ca/HEPES buffer to a final OD_600_ of 0.024. Aliquots of 50 µL of this *E. coli* suspension and 50 µL of a fresh YPSC broth were added to the wells of these plates and the blank measured at 600 nm. Final OD_600_ measurements were carried out after an incubation of 24 hours at 29 °C under shaking conditions. Data is shown as percentage of growth and plotted in a graph against the logarithm of antibiotic concentration. MIC values were calculated by fitting data with a Gompertz modified equation^15^ (using GraphPad Prism 5).

### Agar plate dilution assays

*E. coli* BW25113ΔacrB and *E. coli* MC1061 were transformed within one week prior to the assay, with either vectors derived from p7XC3H and pBXKCPD bearing *folA* variants, respectively. Overnight cultures were grown in LB broth supplemented with the appropriate antibiotic marker and then diluted to the OD_600_ of 1 with the fresh broth. Five µL of two-fold serial dilutions of these cultures were spotted onto 1.5% (w/v) agar Müller-Hinton II (MHII; Roth) plates supplemented with the antibiotic marker and TMP at different concentrations. The plates used to test *E. coli* BW25113ΔacrB bearing p7XC3H derived vectors were supplemented with 0.01 mM IPTG, while the plates used to test *E. coli* MC1061 bearing pBXKCPD derived vectors were supplemented with 0.05% (w/v) L-arabinose. Plates were incubated upside-down at 37 °C for 18–20 hours. The expression of each DHFR variant in control plates was assessed by means of SDS-PAGE and Western blot analysis.

### MIC assays of *E. coli* expressing Bd DHFR variants

*E. coli* BW25113ΔacrB was transformed with either p7XC3H-derived vectors bearing a *folA* gene or the empty vector p7XC3HΔ(ccdB-cmR), within one week prior to the assay. Two-fold serial dilutions of TMP (in MHII broth supplemented with 50 µg/mL kanamycin and 0.01 mM IPTG) were prepared along the rows of 96-well plates (BRANDplates pureGrade^TM^ S, no. 781662). Subsequently, cells from overnight cultures were added to the wells to a final OD_600_ of 0.006 and a final volume of 200 µL. At this point, which corresponds to time zero, the blank was determined by a measurement of the OD_600_ of cell suspensions in a plate reader (TECAN Infinite 200 Pro). Final OD_600_ measurements were carried out as quadruplicates after an incubation of 20 hours at 37 °C under shaking conditions. MIC values were calculated by fitting data with a Gompertz modified equation^15^ (using GraphPad Prism 5).

### DHFR expression and purification

*E. coli* MC1061 transformed with pBXKCPD-derived vectors bearing the *folA* variants *b0048*, *bd3231*, *bd3231*_M29L, *bd3231*_F51I and *bd3231*_M29L_F51I were grown in Terrific Broth supplemented with 50 µg/mL kanamycin. Growth of cultures was started by using a 1% (v/v) overnight culture inoculum and incubation at 37 °C under shaking conditions (200 rpm). Protein expression was induced at the OD_600_ of 0.6 by the addition of 0.05% (w/v) L-arabinose. Cultures were further incubated at 25 °C for 18–20 hours under shaking conditions.

All purification steps of overexpressed proteins explained below were carried out at 4 °C and using filter-sterile (0.2 µm), ice-cold solutions. Cells were pelleted at 15,000 g and washed in cell wash buffer (300 mM NaCl, 20 mM Tris-HCl, pH 8.5 (4 °C), 10 mM imidazole pH 8.5 (4 °C)). Cell pellets were re-suspended in 3 mL lysis buffer (0.1% Triton X-100, 1 mM β-mercaptoethanol, 300 mM NaCl, 20 mM Tris-HCl, pH 8.5 (4 °C), 10 mM imidazole pH 8.5 (4 °C), 10% (v/v) glycerol, 1 mM MgCl_2_, trace amounts of DNAse I, 1 mM PMSF, 1 mg/ml lysozyme) per g of wet weight and incubated on ice for 30 minutes. Cells were lysed by sonication and the lysates cleared by centrifugation at 6,000 g for 20 minutes. Cell debris was removed by two low speed centrifugation steps (20 minutes at 6,000 g and 15 minutes at 15,000 g). Supernatant was applied onto a Ni-NTA Sepharose column (Qiagen). Subsequently, the flow-through was collected and the column washed with wash buffer (0.1% Triton X-100, 1 mM β-mercaptoethanol, 300 mM NaCl, 20 mM Tris-HCl pH 8.5 (4 °C), 30 mM imidazole pH 8.5 (4 °C) and 10% (v/v) glycerol). All DHFR variants were eluted from columns by the addition of 10 mL elution buffer (0.1% (v/v) Triton X-100, 1 mM β-mercaptoethanol, 300 mM NaCl, 20 mM Tris-HCl pH 8.5 (4 °C), 10 mM imidazole pH 8.5 (4 °C), 10% (v/v) glycerol, 5 µM NADPH and 200 µM inositol hexakisphosphate) per bed volume after an overnight incubation (>16 hours) in batch with mixing by rotary inversion. Eluates were concentrated using Amicon Ultra centrifugal filters with a cut-off of 10 kDa. The concentrated solutions were centrifuged for 20 minutes at 16,000 g to remove insoluble aggregates, after which the supernatant was flash frozen in liquid nitrogen and stored at −80 °C until use.

The Bd DHFR wild type variant was further purified via size-exclusion chromatography using a Superdex 75 column coupled to a FPLC (ÄKTA purifier) with a flow rate of 0.7 mL/min and a mobile phase containing 0.1% (v/v) Triton X-100, 1 mM β-mercaptoethanol, 300 mM NaCl, 20 mM Tris-HCl, 10 mM imidazole, 10% (v/v) glycerol, 5 µM NADPH, adjusted to pH 8.5 at 4 °C, 0.2 µm filter sterilized and degassed.

### DHFR *in vitro* assays

DHFR activities were determined in a plate reader (TECAN Infinite 200 Pro) by measuring the decrease in absorbance at 340 nm at 25 °C. Measurements were performed as triplicates in clear 96-well plates (BRANDplates pureGrade^TM^ S, no. 781662) every 15 s, over a time of 10 minutes in the presence of assay buffer (20 mM Tris-HCl, 150 mM NaCl, 0.1 mM EDTA, 1 mM β-mercaptoethanol, pH 7.5 at 25 °C). The conversion of substrate into product was quantified using an extinction coefficient for the reaction of 12.3 mM^−1^ cm^−1^ ^32^. The concentrations of DHF and NADPH were determined spectroscopically using the extinction coefficients of 28,000 M^−1^ cm^−1^ at 282 nm and 6,200 M^−1^ cm^−1^ at 339 nm, respectively. The concentration of active enzymes was determined by titration with methotrexate^33^. The hysteresis effect (slow change in reaction velocity) was prevented by concentrating the DHFR enzymes in the master mix solutions 10 times their working concentrations^34^.

The kinetic values *K*_*m*_ (the Michaelis-Menten constant relative to the substrate, DHF) and *V*_*max*_, for all DHFR enzymes, were determined via kinetic assays in which the concentration of co-substrate, NADPH, was kept constant in all reactions at a saturating level while the substrate, DHF, was tested at different concentrations. Initial velocities were plotted in a graph against substrate concentrations and fitted with the “Substrate inhibition” equation (GraphPad Prism 5; Fig. S7): *v*_*0*_ = (*V*_*max*_[*S*])/(*K*_*m*_ + [*S*]((1 + [*S*])/*K*_*i(sub)*_); where *v*_*0*_ is the initial velocity, *V*_*max*_ the maximum velocity, [*S*] the concentration of substrate, *K*_*m*_ the Michaelis-Menten constant for the substrate and *K*_*i(sub)*_ the substrate dissociation constant. The turnover number, *K*_*cat*_, was calculated by the equation: *K*_*cat*_ = *V*_*max*_/[*Et*]; where [*Et*] is the total enzyme concentration.

TMP inhibition of DHFR activities was tested via inhibition assays in which both NADPH and DHF concentrations were kept constant while the concentration of inhibitor was varied in each reaction.

Data were converted to *v*_*i*_/*v*_*0*_ values (where *v*_*i*_ is the initial velocity of inhibited reactions) and fitted to the following equation: *y* = 1/(1 + [TMP]/*IC*_50_. (GraphPad Prism 5; “log(inhibitor) vs. response” equation with top and bottom values constrained to 1 and 0, respectively; Fig. S8). TMP inhibition constant values (*K*_*i*_) were calculated with the Cheng-Prusoff equation for competitive inhibition^35^: *K*_*i*_ = *K*_*i*_^*app*^/(1 + ([*S*]/*K*_*m*_)); where *K*_*m*_ is the Michaelis-Menten constant for the substrate (DHF) obtained from kinetic experiments, [*S*] the substrate concentration and *K*_*i*_^*app*^ the apparent *K*_*i*_, which here is equal to the *IC*_50_.

## Supplementary information

Supplementary Information.
