# Supplementary material for: A novel method to determine antibiotic sensitivity in Bdellovibrio bacteriovorus reveals a DHFR-dependent natural trimethoprim resistance
Source: Sci Rep. 2020 Mar 24;10:5315. doi: 10.1038/s41598-020-62014-x (PMC7093396; doi:10.1038/s41598-020-62014-x)
Supplement: Supplementary file 1 — Supplementary Information. [file 41598_2020_62014_MOESM1_ESM.pdf]

Supplementary Information:

A novel method to determine antibiotic sensitivity in *Bdellovibrio bacteriovorus* reveals a DHFR-dependent natural trimethoprim resistance

Emanuele Marine[1], David Stephen Milner[2,3], Carey Lambert[2], Renee Elizabeth Sockett[2] & Klaas Martinus Pos[1]*

[1]Institute of Biochemistry, Goethe-University Frankfurt, D-60438, Frankfurt am Main, Germany. [2]Institute of Immunology and Microbiology, School of Life Sciences, University of Nottingham, Queen's Medical Centre, Nottingham, NG7 2UH, UK. [3]Present address: Department of Zoology, University of Oxford, 11a Mansfield Road, Oxford, OX1 3SZ, UK.

*correspondence to: pos@em.uni-frankfurt.de

Table S1 Growth media used in this study.

**Media**	**Components**
Lysogeny broth (LB)	10 g/L Tryptone (Bacto™, BD)<br>5 g/L Yeast extract (Bacto™, BD)<br>5 g/L NaCl
Müller-Hinton II (MHII; Roth)	2g/L Beef Infusion<br>17.5 g/L Casein peptone (acidic hydrolysate)<br>1.5 g/L Corn starch<br>pH 7.4
Terrific broth (TB)	12 g/L Tryptone (Bacto™, BD)<br>24 g/L Yeast extract (Bacto™, BD)<br>5 g/L Glycerol<br>10% (v/v) Phosphate salt solution<br>Added after autoclaving and prior to use<br>Phosphate salt solution: 0.17 M KH_2PO_4 0.72 M K_2HPO_4
YPSC broth	1 g/L Yeast extract (Bacto™, BD)<br>1 g/L Peptone (Bacto™, BD)<br>0.5 g/L Anhydrous sodium acetate<br>0.25 g/L $MgSO_4 \cdot 7H_2O$<br>Adjusted to pH 7.6 with NaOH<br>Supplemented with 0.25 g/L of $CaCl_2$ from a stock of 25 g/L, after autoclaving

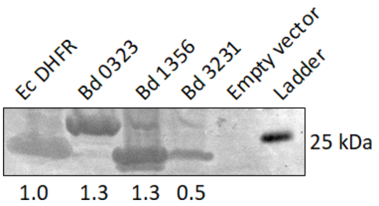

Figure S1 Western blot analysis of cells taken from the control plate (without TMP) in Figure 3 showing band intensities normalized on Ec DHFR expression levels.

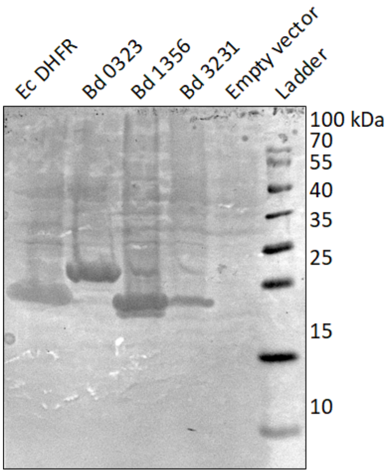

Figure S2 Uncropped Western blot from Figure S1

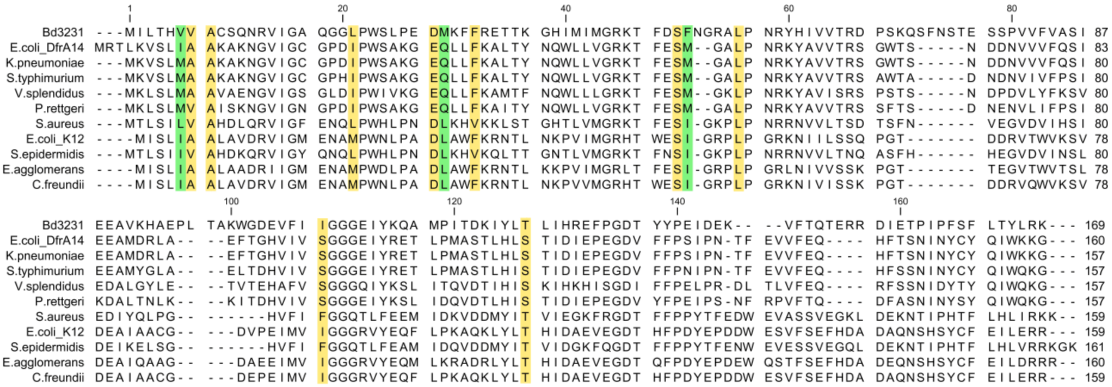

Figure S3 Amino acid sequence alignment of *B. bacteriovorus* DHFR (Bd3231) with trimethoprim sensitive (*Staphylococcus aureus* ATCC 12600, *Escherichia coli* K-12, *Staphylococcus epidermidis* ATCC 14990, *Enterobacter agglomerans* ATCC 27155, *Citrobacter freundii* ATCC 8090) and resistant DHFRs (*Escherichia coli* ATCC 11775 (DfrA14), *Klebsiella pneumoniae* ATCC 13883, *Salmonella typhimurium* ATCC 13311, *Vibrio splendidus* ATCC 33125, *Providencia rettgeri* ATCC 29944). Amino acids highlighted in green relate to positions involved in trimethoprim binding which are occupied by different amino acids in *B. bacteriovorus* DHFR in comparison to TMP-sensitive species. Amino acids highlighted in yellow relate to positions involved in trimethoprim binding which are occupied by the same amino acids in *B. bacteriovorus* DHFR in comparison to TMP-sensitive species. Positions involved in trimethoprim binding were taken from *E. coli* K-12 DHFR binding studies[1].

Table S2 Amino acidic sequence homology of *B. bacteriovorus* DHFR for DHFRs from TMP-sensitive and TMP-resistant bacteria. Values were obtained by multiple alignments using Clustal Omega (EMBL-EBI) and are expressed as percentage of identity, similarity and gaps extension.

		B. bacteriovorus	**DfrA14**	***K. pneumoniae***	***P. rettgeri***	***S. typhimurium***	***V. splendidus***	***C. freundii***	***E. agglomerans***	***E. coli*_K12**	***S. aureus***	***S. epidermidis***
B. bacteriovorus	Identity	100%										
	Similarity	0%										
	Gaps	0%										
**DfrA14**	Identity	28%	100%									
	Similarity	48%	0%									
	Gaps	13%	0%									
K. pneumoniae	Identity	30%	96%	100%								
	Similarity	49%	98%	0%								
	Gaps	11%	1%	0%								
P. rettgeri	Identity	34%	70%	72%	100%							
	Similarity	53%	83%	84%	0%							
	Gaps	11%	1%	0%	0%							
S. typhimurium	Identity	29%	87%	90%	75%	100%						
	Similarity	50%	94%	96%	87%	0%						
	Gaps	11%	1%	0%	0%	0%						
V. splendidus	Identity	28%	58%	61%	65%	60%	100%					
	Similarity	52%	75%	77%	82%	77%	0%					
	Gaps	11%	1%	0%	0%	0%	0%					
C. freundii	Identity	30%	33%	33%	27%	32%	30%	100%				
	Similarity	52%	53%	53%	54%	53%	52%	0%				
	Gaps	10%	7%	6%	6%	6%	6%	0%				
E. agglomerans	Identity	31%	29%	29%	27%	29%	26%	80%	100%			
	Similarity	52%	47%	48%	51%	50%	50%	88%	0%			
	Gaps	10%	8%	6%	6%	6%	6%	0%	0%			
***E. coli*_K12**	Identity	30%	33%	33%	26%	31%	28%	96%	80%	100%		
	Similarity	52%	53%	53%	54%	53%	52%	98%	89%	0%		
	Gaps	10%	7%	6%	6%	6%	6%	0%	0%	0%		
S.aureus	Identity	33%	25%	26%	28%	26%	27%	34%	35%	33%	100%	
	Similarity	52%	44%	45%	50%	44%	45%	56%	58%	56%	0%	
	Gaps	11%	8%	7%	7%	7%	7%	3%	3%	3%	0%	
S.epidermidis	Identity	31%	25%	25%	28%	27%	25%	34%	37%	34%	80%	100%
	Similarity	52%	42%	43%	49%	44%	44%	54%	57%	54%	93%	0%
	Gaps	12%	10%	8%	8%	8%	8%	4%	4%	4%	1%	0%

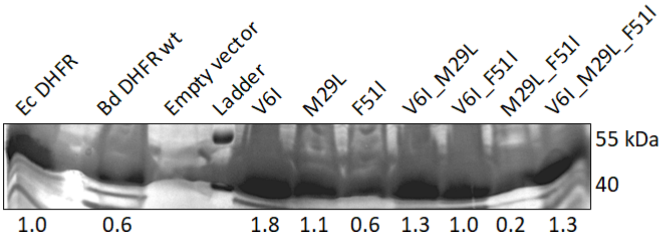

Figure S4 Western blot analysis of cells taken from the control plate (without TMP) in Figure 5 showing band intensities normalized on Ec DHFR expression levels.

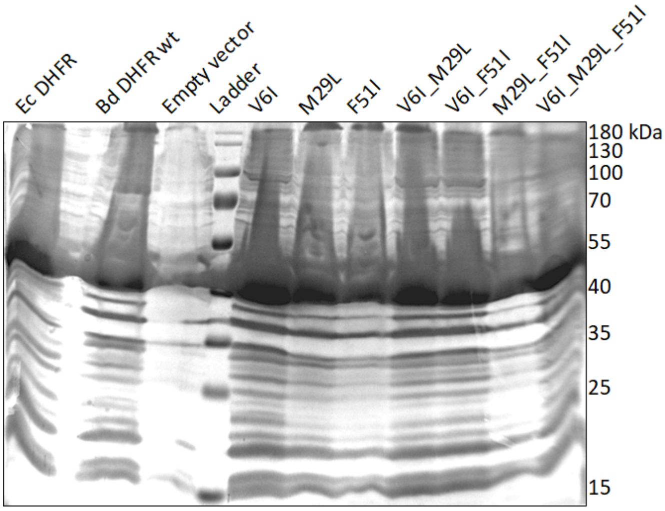

Figure S5 Uncropped Western blot from Figure S4

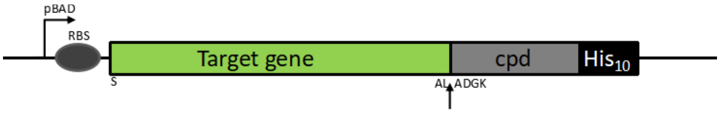

Figure S6 Detail of the cloning site of the pBXKCPD vector after cloning a target gene via fragment exchange (FX-cloning[2]). The target protein bears an additional serine at the N-terminus as the 2nd amino acid of its sequence and an alanine at the C-terminus, as a result of the cloning process. The ALADGK sequence (the first alanine being a leftover of the FX-cloning procedure) is cleaved (arrow) by the CPD protease to leave only an alanine and a leucine attached to the target protein. Cleavage of recombinant proteins is triggered by the addition of inositol hexakisphosphate directly in the affinity column, allowing purified proteins to be eluted in a single step without the CPD-tags (which are retained in the columns)[3].

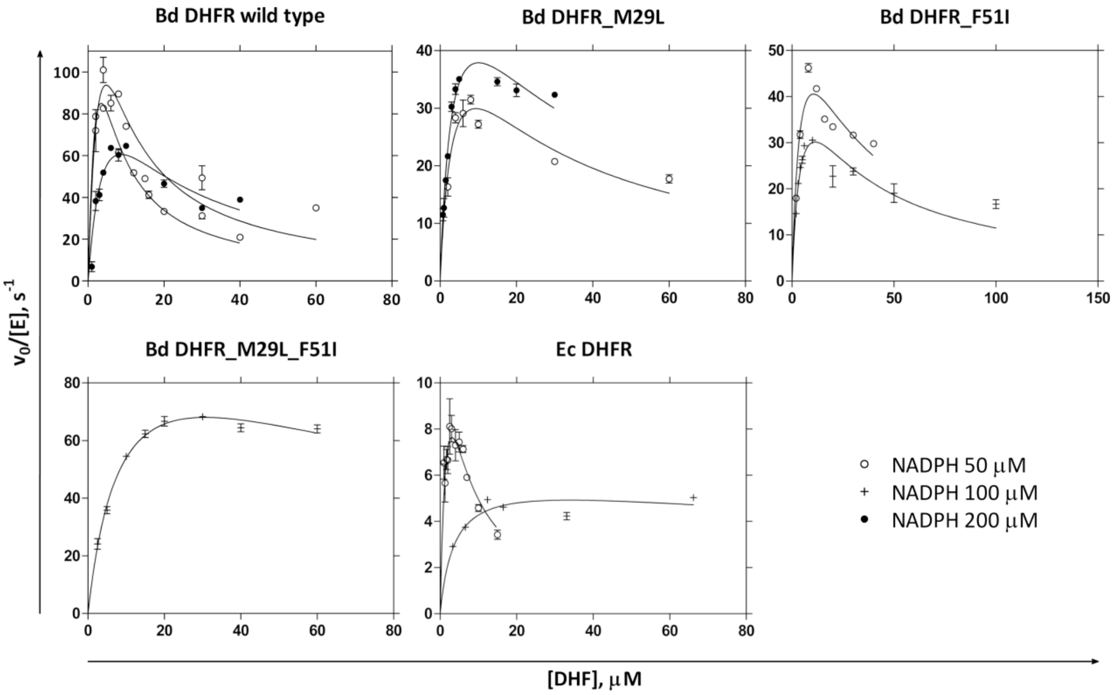

Figure S7 Kinetic studies on purified DHFR. Each data point shown is the mean of triplicate determinations. Data were fitted with the "Substrate inhibition" equation (GraphPad Prism 5).

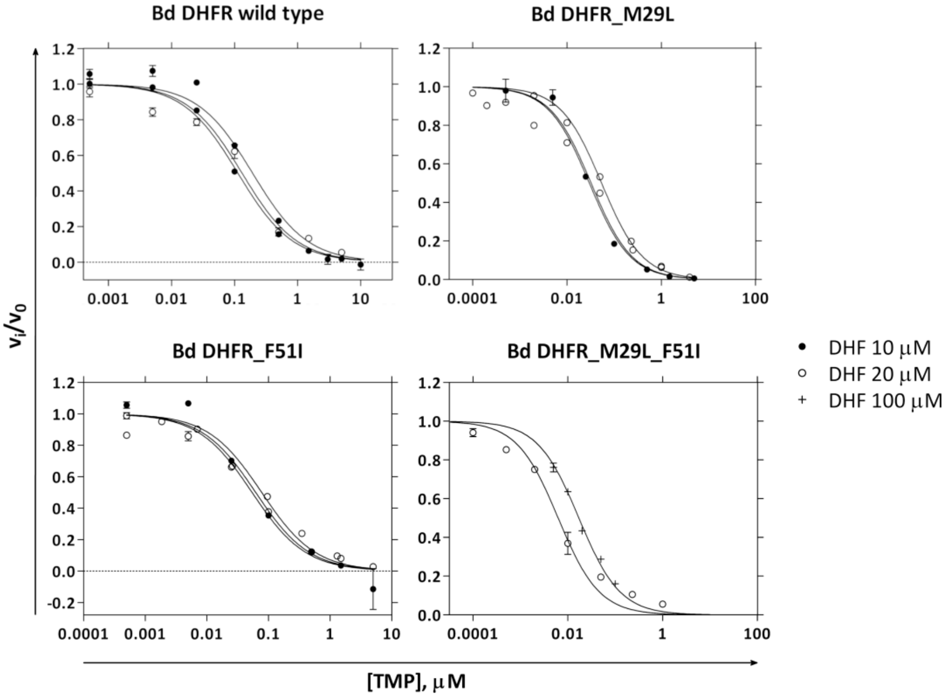

Figure S8 TMP inhibition of DHFRs activities. Each data point shown is the mean of triplicate determinations. Experiments were performed at different DHF concentrations and in presence of either 60 μM (Bd DHFR_M29L_F51I at 100 μM DHF) or 200 μM NADPH. Normalized data were fitted with a logistic equation ("log(inhibitor) vs. response" equation by constraining Top and Bottom values to 1 and 0, respectively; GraphPad Prism 5).

Table S3 Bacterial strains used in this study.

Strain	Description	Source	Reference
E. coli BW25113	*F^- Δ(araD-araB)567 ΔlacZ4787(::rrnB-3) λ^- rph-1 Δ(rhaD-rhaB)568 hsdR514*	Laboratory collection	Datsenko and Wanner, 2000[4]; Grenier *et al.*, 2014[5]
E. coli BW25113ΔacrAB	*F^- Δ(araD-araB)567 ΔlacZ4787(::rrnB-3) λ^- rph-1 Δ(rhaD-rhaB)568 hsdR514 ΔacrAB*	Laboratory collection	Lytvynenko *et al.*, 2016[6]
E. coli DB3.1	*F^- gyrA462 endA1 Δ(sr1-recA) mcrB mrr hsdS20 supE44 ara14 galK2 lacY1 proA2 rpsL20 xyl5 leuB6 mtl1*	Geertsma E, Frankfurt University	Bernard and Couturier, 1992[7]
E. coli MC1061	*str. K-12 F^- λ^- Δ(araA-leu)7697 [araD139]B/r Δ(codB-lacI)3 galK16 galE15 e14– mcrA0 relA1 rpsL150(StrR) spoT1 mcrB1 hsdR2(r–m+)*	Geertsma E, Frankfurt University	Casadaban and Cohen, 1980[8]
B. bacteriovorus HD100	Wild type	Sockett RE, Queen's Medical Centre Nottingham	Stolp and Starr, 1963[9]; Rendulic *et al.*, 2004[10]

Table S4 List of plasmids used in this study.

Plasmid name	Description	Source	Reference
p7XC3H	It contains a kanamycin resistance marker and a FX-cloning site preceded by the T7 promoter/*lac* operator and a ribosome binding site and followed by a 3C protease cleavage site and a His_{10}-tag.	Geertsma ER, Frankfurt University	Geertsma and Dutzler, 2011[2]
p7XC3Hb0048	Derivative of p7XC3H containing the *b0048* gene (*folA*) from *E. coli.*	This study	
p7XC3Hbd0323	Derivative of p7XC3H containing the *bd0323* gene from *B. bacteriovorus.*	This study	
p7XC3Hbd1356	Derivative of p7XC3H containing the *bd1356* gene from *B. bacteriovorus.*	This study	
p7XC3Hbd3231	Derivative of p7XC3H containing the *bd3231* gene from *B. bacteriovorus.*	This study	
p7XC3Hbd3231_F51I	Derivative of p7XC3H containing the *bd3231* gene from *B. bacteriovorus* bearing the mutation F51I.	This study	
p7XC3Hbd3231_M29L	Derivative of p7XC3H containing the *bd3231* gene from *B. bacteriovorus* bearing the mutation M29L.	This study	
p7XC3Hbd3231_M29L_F51I	Derivative of p7XC3H containing the *bd3231* gene from *B. bacteriovorus* bearing the mutations M29L and F51I.	This study	
p7XC3Hbd3231_V6	Derivative of p7XC3H containing the *bd3231* gene from *B. bacteriovorus* bearing the mutation V6I.	This study	

p7XC3Hbd3231_V6I_F51I	Derivative of p7XC3H containing the *bd3231* gene from *B. bacteriovorus* bearing the mutations V6I and F51I.	This study	
p7XC3Hbd3231_V6I_M29L	Derivative of p7XC3H containing the *bd3231* gene from *B. bacteriovorus* bearing the mutations V6I and M29L.	This study	
p7XC3Hbd3231_V6I_M29L_F51I	Derivative of p7XC3H containing the *bd3231* gene from *B. bacteriovorus* bearing the mutations V6I, M29L and F51I.	This study	
p7XC3HΔ(ccdB-cmR)	Derivative of p7XC3H with deletion of the sequence comprised between the two SapI restriction sites that includes the two genes *ccdB* and *cmR.*	This study	
pBXKCPD	It contains a kanamycin resistance marker, a FX-cloning site preceded by a P_{BAD} promoter and a ribosome binding site and followed by a CPD-tag fused with a His_{10}-tag.	This study	
pBXKCPDb0048	Derivative of pBXKCPD containing the *b0048* gene (*folA*) from *E. coli.*	This study	
pBXKCPDbd3231	Derivative of pBXKCPD containing the *bd3231* gene from *B. bacteriovorus.*	This study	
pBXKCPDbd3231_F51I	Derivative of pBXKCPD containing the *bd3231* gene from *B. bacteriovorus* bearing the mutation F51I.	This study	
pBXKCPDbd3231_M29L	Derivative of pBXKCPD containing the *bd3231* gene from *B. bacteriovorus* bearing the mutation M29L.	This study	
pBXKCPDbd3231_M29L_F51I	Derivative of pBXKCPD containing the *bd3231* gene from *B. bacteriovorus* bearing the mutations M29L and F51I.	This study	
pBXKCPDbd3231_V6I	Derivative of pBXKCPD containing the *bd3231* gene from *B. bacteriovorus* bearing the mutation V6I.	This study	
pBXKCPDbd3231_V6I_F51I	Derivative of pBXKCPD containing the *bd3231* gene from *B. bacteriovorus* bearing the mutations V6I and F51I.	This study	
pBXKCPDbd3231_V6I_M29L	Derivative of pBXKCPD containing the *bd3231* gene from *B. bacteriovorus* bearing the mutations V6I and M29L.	This study	
pBXKCPDbd3231_V6I_M29L_F51I	Derivative of pBXKCPD containing the *bd3231* gene from *B. bacteriovorus* bearing the mutations V6I, M29L and F51I.	This study	
pBXKCPDΔ(ccdB-cmR)	Derivative of pBXKCPD with deletion of the sequence comprised between the two SapI restriction sites that includes the two genes *ccdB* and *cmR.*	This study	

pET21pto0987cpd	Derivative of pET21 containing the *pto0987* gene from *Picrophilus torridus* fused to a cpd-tag.	Laboratory collection	
pINITcat	It contains a chloramphenicol resistance marker and a FX-cloning site (SapI restriction sites oriented toward the exchanged fragment) NOT preceded by a promoter (sequencing plasmid) and a ribosome binding site.	Geertsma ER, Frankfurt University	Geertsma and Dutzler, 2001[2]
pINITcatb0048	Derivative of pINITcat containing the *b0048* gene (*folA*) from *E. coli.*	This study	
pINITcatbd0323	Derivative of pINITcat containing the *bd0323* gene from *B. bacteriovorus.*	This study	
pINITcatbd1356	Derivative of pINITcat containing the *bd1356* gene from *B. bacteriovorus.*	This study	
pINITcatbd3231	Derivative of pINITcat containing the *bd3231* gene from *B. bacteriovorus.*	This study	
pINITcatbd3231_F51I	Derivative of pINITcat containing the *bd3231* gene from *B. bacteriovorus* bearing the mutation F51I.	This study	
pINITcatbd3231_M29L	Derivative of pINITcat containing the *bd3231* gene from *B. bacteriovorus* bearing the mutation M29L.	This study	
pINITcatbd3231_M29L_F51I	Derivative of pINITcat containing the *bd3231* gene from *B. bacteriovorus* bearing the mutations M29L and F51I.	This study	
pINITcatbd3231_V6I	Derivative of pINITcat containing the *bd3231* gene from *B. bacteriovorus* bearing the mutation V6I.	This study	
pINITcatbd3231_V6I_F51I	Derivative of pINITcat containing the *bd3231* gene from *B. bacteriovorus* bearing the mutations V6I and F51I.	This study	
pINITcatbd3231_V6I_M29L	Derivative of pINITcat containing the *bd3231* gene from *B. bacteriovorus* bearing the mutations V6I and M29L.	This study	
pINITcatbd3231_V6I_M29L_F51I	Derivative of pINITcat containing the *bd3231* gene from *B. bacteriovorus* bearing the mutations V6I, M29L and F51I.	This study	

Construction of *E. coli* strains. Primers used in this study are listed in Table S5.

The expression vector pBXKCPD compatible with the FX-cloning system was derived from the vector pBXC3H[2], by replacing the ampicillin cassette with a kanamycin resistance gene, deleting the 3C protease cleavage site and inserting the *cpd* sequence (cysteine protease domain of the *Vibrio cholerae* MARTX toxin[3]) preceded by its cleavage site, between the His_{10}-tag and the adjacent SapI site. The *cpd* sequence was amplified from plasmid DNA (pET21pto0987cpd) by PCR using the primers #1 and #2. This fragment and the plasmid pBXC3H were double digested with SpeI and XbaI and then, ligated. The kanamycin resistance gene from another FX-cloning compatible expression vector (p7XC3H[2]) was amplified by PCR using the primers #3 and #4, while the plasmid backbone was amplified using the primers #5 and #6. The two fragments were mixed together and joined by PCR using the primers #3 and #5, the resulting stretch of DNA was digested with PagI and circularized by ligation. The *E. coli* DB3.1 strain was used for the construction, amplification and maintenance of this vector since it is resistant to the toxic protein encoded by the *ccdB* gene (i.e. counter-selection marker).

Variants of the FX-cloning plasmids p7XC3H and pBXKCPD, devoid of the *ccdB-cmR* fragment, were constructed in order to be used as negative controls in different experiments. The original plasmids were digested with SapI and the backbones fragments gel purified. Single-stranded overhangs at both 3’ and 5’ ends were degraded by Mung bean nuclease (New England Biolabs) digestion then, fragments were purified and circularized by T4 ligase blunt-end ligation.

The genes *bd3231*, *bd0323*, *bd1356* were amplified from *B. bacteriovorus* genomic DNA by PCR using the dedicated pairs of primers (#7 – #8, #9 – #10, #11 – #12, respectively). The gene *b0048* was amplified from *E. coli* BW25113 genomic DNA by PCR using the primers #13 and #14. The *folA* gene from *B. bacteriovorus* (*bd3231*) was mutated by site-directed mutagenesis. The primers #15 and #16 were used to introduce the mutation V6I, while the mutation M29L was introduced with the primers #17 and #18 and the mutation F51I with the primers #19 and #20. All genes were cloned into the FX-cloning sequencing vector pINITcat[2] and then sub-cloned into pBXKCPD and/or p7XC3H[2]. The *E. coli* strain MC1061 was employed for all steps of cloning and amplification of vectors bearing these genes.

Table S5 Oligonucleotide primers used in this study.

#	Primer name	Sequence
1	SpeI cpd tag fw	ATATACTAGTTCAGCTCTTCTGCATTGGCAGACGGCAAGATTCT
2	XbaI cpd his10 rv	ATATTCTAGATTATTAATGATGATGATGATGGTGATGATGATGGTGACCCTGCGCATCCCAAGACA
3	KmR p7X fw	TTTGTTTATTTTTCTAAATACATTCAAATATGTATCCGCTCATGA
4	KmR p7X rv	CTACGGGGTCTGACGCTCAGTGG
5	KmR pBX fw	CCACTGAGCGTCAGACCCCGTAG
6	KmR pBX rv	TCATGAGCGGATACATATTTGAATGTATTTAGAAAAATAAACAAA
7	FX bd3231 fw	TATATGCTCTTCTAGTATTTTGACTCATGTGGTGGCCTG
8	FX bd3231 rv	TATATGCTCTTCATGCCTTGCGCAAGTAAGTCAGAAACG
9	FX bd0323 fw	TATATGCTCTTCTAGTACAAATTGCAGGGTTTCAAAACCC
10	FX bd0323 rv	TATATGCTCTTCATGCGTGAAGGTCGTCGTGAATATCC
11	FX bd1356 fw	TATATGCTCTTCTAGTCCGCTGCATCGAAACTATGG
12	FX bd1356 rv	TATATGCTCTTCATGCCGTGGTAGCTATTGAATAAATGACC
13	FX b0048 fw	TATATGCTCTTCTAGTATCAGTCTGATTGCGGCGTTAG
14	FX b0048 rv	TATATGCTCTTCATGCCCGCCGCTCCAGAATCTCAAAG
15	bd3231 V6I fw	TTTGACTCATATTGTGGCCTGTTC
16	bd3231 V6I rv	ATACTCATATGTATATCTCCTTC
17	bd3231 M29L fw	GCCCGAGGACCTGAAGTTCTTCC
18	bd3231 M29L rv	AGACTCCAGGGAAGGCCC
19	bd3231 F51I fw	GTTTGATTCTATCAACGGCCG
20	bd3231 F51I rv	GTCTTGCGCCCCATGATC

Table S6 List of antibiotics used in this study.

Compound	Formulation	Supplier	Concentration (mg/ml)	Solvent
Amikacin	$2{\cdot}H_2SO_4$	Alfa Aesar	50	H_2O
Ampicillin	Na^+	Roth	100	H_2O
Aztreonam		Alfa Aesar	50	DMF:methanol (1:1)
Cefaclor		Sigma-Aldrich	2	Ca/HEPES buffer
Cefazolin	Na^+	Alfa Aesar	50	H_2O
Cefoperazone		Alfa Aesar	50	H_2O
Ceftazidime		Molekula	50	H_2O
Chloramphenicol		Applichem	30	Isopropanol
Ciprofloxacin	HCl	Applichem	20	H_2O
Gentamicin	H_2SO_4	Applichem	20	H_2O
Kanamycin	H_2SO_4	Roth	50	H_2O
Levofloxacin		Fluka	2	H_2O
Meropenem		Molekula	20	DMSO
Penicillin G	Na^+	Fluka	100	H_2O
Piperacillin	Na^+	Sigma-Aldrich	10	H_2O
Tetracycline	HCl	Applichem	10	H_2O
Tigecycline		Calbiochem	30	DMSO
Trimethoprim		Fluka	25	DMF:methanol (1:1)

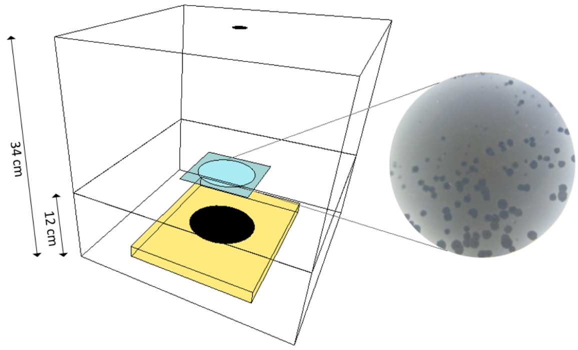

Figure S9 Plaques Visualization Chamber design. A piece of black cardboard (Ø 11.5 cm) is positioned on the top of a transilluminator (in yellow) with its center perpendicular to the hole covered with glass (in light blue; Ø 9 cm). Plates were placed above the glass and their pictures taken through the hole on the top of the chamber.

References

1. Matthews, D. A. *et al.* Refined crystal structures of Escherichia coli and chicken liver dihydrofolate reductase containing bound trimethoprim. *J. Biol. Chem.* **260**, 381–391 (1985).
2. Geertsma, E. R. & Dutzler, R. A versatile and efficient high-throughput cloning tool for structural biology. *Biochemistry* **50**, 3272–3278 (2011).
3. Shen, A. *et al.* Simplified, enhanced protein purification using an inducible, autoprocessing enzyme tag. *PLoS One* **4**, (2009).
4. Datsenko, K. A. & Wanner, B. L. One-step inactivation of chromosomal genes in Escherichia coli K-12 using PCR products. *Proc. Natl. Acad. Sci.* **97**, 6640–6645 (2000).
5. Grenier, F., Matteau, D., Baby, V. & Rodrigue, S. Complete Genome Sequence of Escherichia coli BW25113. *Genome Announc.* **2**, 90005 (2014).
6. Lytvynenko, I., Brill, S., Osvald, C. & Pos, K. M. Molecular basis of polyspecificity of the Small Multidrug Resistance Efflux Pump AbeS from Acinetobacter baumannii. *J. Mol. Biol.* **428**, 644–657 (2016).
7. Bernard, P. & Couturier, M. Cell killing by the F plasmid CcdB protein involves poisoning of DNA-topoisomerase II complexes. *J. Mol. Biol.* **226**, 735–45 (1992).
8. Casadaban, M. J. & Cohen, S. N. Analysis of gene control signals by DNA fusion and cloning in Escherichia coli. *J. Mol. Biol.* **138**, 179–207 (1980).
9. Stolp, H. & Starr, M. P. Bdellovibrio bacteriovorus gen. et sp. n., a predatory, ectoparasitic, and bacteriolytic microorganism. *Antonie Van Leeuwenhoek* **29**, 217–248 (1963).
10. Rendulic, S. *et al.* A Predator Unmasked: Life Cycle of Bdellovibrio bacteriovorus from a Genomic Perspective. *Science (80-.).* **303**, 689–692 (2004).
